# Does bilateral Wilms’ tumor involving the collecting system in children have a worse prognosis?

**DOI:** 10.1186/s12894-024-01525-5

**Published:** 2024-07-08

**Authors:** YiWei Fang, ZhenWu Li, HongCheng Song, WeiPing Zhang, Ning Li, Yang Yang

**Affiliations:** Department of Urology, Beijing Children’s Hospital, Capital Medical University, National Center for Children’s Health, No. 56 Nanlishi St, Xicheng District, Beijing, 100045 China

**Keywords:** Nephron-sparing surgery, Bilateral wilms’ tumor, Collecting system

## Abstract

**Background:**

The literature on nephron-sparing surgery (NSS) in children with bilateral Wilms’ tumors (BWT) involving the collection system is mostly comprised of case reports. The present study aimed to summarize the clinical characteristics, treatments, and prognosis of children with BWT involving the collecting system admitted to our pediatric surgery center compared with those whose tumors did not involve the collecting system. A secondary aim was to discuss how to preserve more kidney parenchyma and prevent long-term renal failure under the premise of preventing tumor recurrence.

**Methods:**

Patients with BWT admitted to our pediatric surgery center between January 2008 and June 2022 were reviewed. All included patients were grouped according to the relationship between the tumor and collecting system according to the intraoperative findings. Group I included children with tumor infiltrating the collecting system, group II included children with tumor growing into the collecting system, and group III included children whose tumor did not involve the collecting system. The clinical features, treatments and prognosis of the patients were analyzed.

**Results:**

Seventy patients were enrolled, including 20 patients with 25 sides of tumors infiltrating the collecting system in group I,10 patients with 13 sides of tumors growing into the collecting system in group II, and 40 patients in group III. There was no significant difference in patients age and gender between group I and group II. In total, 20 patients in group I and 9 patients in group II had partial response (PR) after neoadjuvant chemotherapy. In group I, 22 of 25 sides of tumors underwent NSS; in group II, 11 of 13 sides of tumors underwent NSS. During an average follow-up of 47 months, in group I, 6/20 patients relapsed and 2/20 patients died; in group II, 3/10 patients relapsed and 1/10 patient died. There was no significant difference in 4-year overall survival (OS) rate among groups I, II and III (86.36% vs. 85.71%vs. 91.40%, *P* = 0.902).

**Conclusions:**

To preserve renal parenchyma, NSS is feasible for children with BWT involving the collecting system. There was no significant difference in postoperative long-term OS between patients with BWT involving the collecting system and not involving the collecting system.

## Background

In unilateral Wilms’ tumors (WT), total nephrectomy is recommended when the tumor infiltrates or grows into the collecting system [1]. However, in bilateral Wilms’ tumors (BWT), total nephrectomy on one side and partial nephrectomy on the other increases the likelihood of long-term renal failure. The literature on nephron-sparing surgery (NSS) in children with BWT involving the collection system is mostly comprised of case reports [2]. The present study aimed to summarize the clinical characteristics, treatments, and prognosis of children with BWT involving the collecting system admitted to our pediatric surgery center compared with those whose tumors did not involve the collecting system. A secondary aim was to discuss how to preserve more kidney parenchyma and prevent long-term renal failure under the premise of preventing tumor recurrence.

## Methods

### Patients and grouping

After obtaining institutional authorization ([2021]-E-154-R), we conducted a review of medical records of 70 children diagnosed with BWT between January 2008 and June 2022 at a single pediatric surgery center. The requirement for informed consent was waived due to the retrospective nature of the study. Data on sex, age at presentation, presenting symptoms, radiological results, pathological findings, surgery, preoperative and postoperative treatment, and follow-up were reviewed. All children underwent abdominal ultrasonography, enhanced abdominal computed tomography (CT), and chest CT at diagnosis. All patients with preoperative imaging or intraoperative findings of tumors infiltrating or growing into the collecting system were included. The exclusion criteria were incomplete clinical data, lack of surgical treatment, non-WT postoperative pathology, and nephroblastomatosis.

All included patients were grouped according to the relationship between the tumor and collecting system according to the intraoperative findings. Group I included children with intraoperative findings of tumors infiltrating the collecting system; group II included children with tumors growing into the collecting system (botryoid growth pattern, BGP) [3]; and group III included children whose tumors did not involve the collecting system.

### Response to treatment, pathology, and follow-up

Response to chemotherapy was evaluated according to the Response Evaluation Criteria in Solid Tumors. A complete response (CR) was defined as the disappearance of all tumors, with no appearance of new tumors. A partial response (PR) was defined as a reduction ≥ 30% in the sum of the maximum diameters of the tumor. Progressive disease (PD) was defined as the sum of the maximum diameters of the tumor increasing by at least ≥ 20% or the appearance of a new tumor. Stable disease (SD) was defined as the sum of the maximum diameters of the target tumor lesions not shrinking to the level in PR or increasing to the level in PD.

Pathological classification of children who underwent preoperative chemotherapy was performed according to the International Society of Pediatric Oncology (SIOP) pathological classification system. Lymph node biopsy was routinely performed during the operation. The patients were followed up every 3 months from the first to the second year postoperatively, and every 4 months in the third year postoperatively. In the fourth postoperative year, the patients were followed up every 6 months. Follow-up included abdominal ultrasound, abdominal enhanced CT, and chest radiography, and survival outcomes (recurrence or death) were assessed.

### Statistical analysis

The patient data were analyzed using descriptive statistics. Event-free survival (EFS) was defined as the time from the date of presentation to the first documented recurrence or death. Overall survival (OS) was defined as the time from the date of presentation to the date of death. All patients were considered to calculate EFS and OS. EFS and OS were compared between the groups using the Kaplan–Meier method. All analyses were performed using SPSS version 23.0. Statistical significance was set at *P <* 0.05.

## Results

### Characteristics of children with BWT

**Fig. 1:**
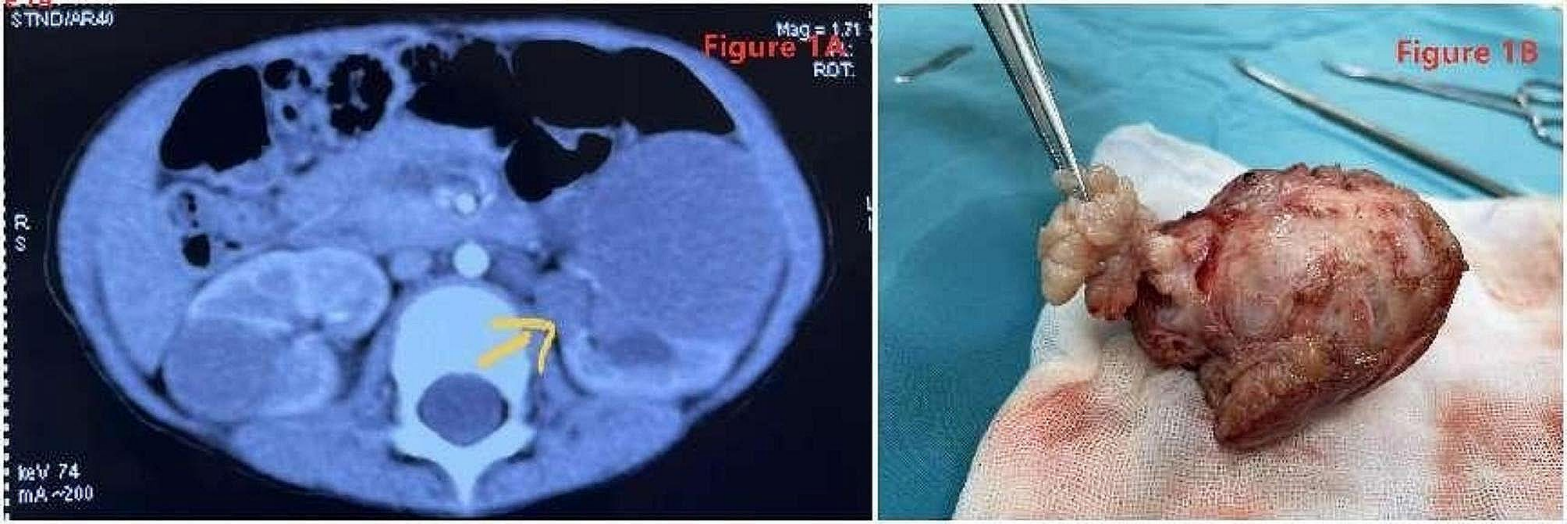
**A** Enhanced CT of bilateral wilms’ tumor with left tumor growing into the renal pelvis. **B** Postoperative gross pathology of the tumor, the renal pelvis mass can be seen intact, showing a grape-like growth pattern

Seventy patients with BWT were admitted to our pediatric surgery center between January 2008 and June 2022. The clinical characteristics of the patients with BWT, including sex, age at presentation, presenting symptoms, and concomitant malformations, are shown in Table 1. Five patients had lung metastases at presentation. Contrast-enhanced CT scans only detected 13 tumor lesions growing into the pelvis, as shown in Fig. 1A.

**Table 1:** Clinical characteristics of children with bilateral Wilms’ tumors by involvement of the collecting system

Variables, *n* (%)	Group I: tumors infiltrating the collecting system (*n*=20)	Group II: tumors growing into the collecting system (*n*=10)	Group III: not involving the collecting system (*n*=40)
Gender			
Male	12	7	20
Female	8	3	20
Age (months)			
Median	17.5	13	12
Range	6–65	8–34	3–75
Classification of BWT			
Synchronous WT	19	10	37
Metachronous WT	1		3
Clinical manifestations			
Abdominal mass	11	7	31
Hematuria	4	2	3
Ultrasound finding	1		4
Abdominal distension	2	1	
Abdominal pain	1		1
Vomiting	1		1
Concomitant malformations			
Cryptorchidism	4	3	5
Hypospadias	3	1	4
Iris agenesis			3
WAGR syndrome	2		3
Indirect inguinal hernia			1
Denys–Drash Syndrome		1	
Other	1		1

Thirty patients (30/70, 42.9%) had tumors involving the collecting system, including 20 patients with 25 kidneys with tumors infiltrating the collecting system in group I and 10 patients with 13 kidneys with tumors growing into the collecting system in group II. There were no significant differences among groups I, II, and III in terms of age or sex (*P* = 0.788 and *P* = 0.471, respectively). The incidence of hematuria was higher in groups I and II than in group III (20% vs. 7.5%, *P =* 0.236); however, the difference was not significant.

### Treatment of children with BWT involving the collecting system

The initial biopsy and preoperative chemotherapy results for groups I and II are shown in Table 2. In group I, 20 patients underwent neoadjuvant chemotherapy, with 23 kidneys (57.5%) achieving PR, 16 kidneys (40.0%) having SD, and 1 kidney (2.5%) having PD. In group II, nine children underwent neoadjuvant chemotherapy, with five kidneys (27.8%) achieving PR, 12 kidneys (66.7%) having SD, and 1 kidney (5.6%) having PD. After preoperative chemotherapy, 13 tumors in the renal pelvis all had SD.

**Table 2:** Treatment of children with bilateral Wilms’ tumor involving the collecting system

	Group I: Infiltrating the collecting system *n* = 20	Group II: Botryoid growth pattern *n* = 10
Preoperative biopsy	4	1
Neoadjuvant chemotherapy		
VA regimen^1^	12	6
VAD regimen^1^	6	2
Unknown regimen	2	1
Duration of neoadjuvant chemotherapy		
Median(weeks)	5	5
Range(weeks)	2–12	2–20
Surgery		
Bilateral NSS	15	7
Nonbilateral NSS^2^	5	3

^1^ VA regimen: vincristine + actinomycin D; VAD regimen: vincristine + actinomycin D + doxorubicin

^2^ Nonbilateral NSS: total nephrectomy for one kidney and NSS for the contralateral kidney

In group I, among the 25 kidneys with tumors infiltrating the collecting system, 3 kidneys underwent total nephrectomy, and 22 kidneys underwent NSS. One child underwent NSS when the tumor infiltrated the collecting system in one kidney had a gross residual tumor at the surgical resection margin (SRM) during NSS. The remaining 21 kidneys showed no gross residual tumor. In group II, among the 13 kidneys with tumors growing into the collecting system, 2 kidneys underwent total nephrectomy, and 11 kidneys underwent NSS in which the tumor and the mass in the collecting system were removed completely without any rupture (Fig. 1B).

### Pathology, postoperative chemotherapy, and radiotherapy

According to the SIOP pathological classifcation system, 20 patients (40 kidneys) with preoperative chemotherapy in Group I had the following postoperative pathologies: 15 kidneys of stromal type, 5 kidneys of epithelial type, 1 kidney of blastemal type, 8 kidneys of mixed type, nephroblatoma completely necrotic in 10 kidneys, fetal rhabdomyomatous nephroblastoma in 1 kidney and no anaplasia type. 9 patients (18 kidneys) with preoperative chemotherapy in Group II had the following postoperative pathologies: 12 kidneys of stromal type, 1 kidney of epithelial type, 3 kidneys of mixed type, nephroblatoma completely necrotic in 2 kidneys and no anaplasia type. In group I, the renal hilar lymph nodes were positive on only one side of the WT. The pathological resection margin (PRM) was positive in 1 of the 35 kidneys that underwent NSS. In group II, the postoperative pathology of one patient who did not undergo neoadjuvant chemotherapy was predominantly mixed in one kidney and predominantly stromal in the other kidney. In group II, the lymph nodes were negative for tumor. The PRM was positive on one kidney.

The postoperative treatment plan was formulated according to the staging criteria for the higher unilateral stage. Patients who received neoadjuvant chemotherapy were treated according to the SIOP staging standard and those who did not receive preoperative chemotherapy were treated according to the National Wilms Tumor Study/Children’s Oncology Group (NWTS/COG) protocol. Only one patient in group II was treated according to the NWTSG/COG protocol. In groups I and II, eight kidneys had radiotherapy, including one kidney with a positive SRM and one kidney with a positive PRM.

### Prognosis

The mean follow-up duration was 47 months (range, 2–162 months). Overall, 16 patients experienced recurrence, and 8 died. Among the eight patients who died, the specific causes of death were cerebral hernia at 9 years without tumor (one case), pulmonary metastasis (two cases), renal failure caused by ESRD (two cases, including one case of Denys–Drash syndrome [DDS]), and tumor recurrence after ineffective surgical treatment (three cases). There were significant differences in the postoperative event-free survival of the 70 children with different tumor stages (92.3% vs. 64% vs. 50% vs. 40%, *P* = 0.005) (Table 3). The 4-year EFS of all patients was 67.9% and the 4-year OS was 89.3%.

**Table 3:** Effect of tumor stage on prognosis

	total (*n*)	Postoperative event-free survival(*n*)	Postoperative recurrence, death(*n*)
Stage I^*^	26	24	2
Stage II^*^	25	16	9
Stage III^*^	14	7	7
Stage IV^*^	5	2	3

^*By the highest stage (either favorable histology focal or diffuse anaplasia present)^

In group I, six patients experienced recurrence, and two patients died after recurrence. Of the six patients with recurrence, five underwent bilateral NSS, and the side of tumor recurrence in four patients was consistent with the side where the tumor infiltrated the collecting system before surgery. All six children underwent surgical resection after tumor recurrence and additional chemotherapy was administered postoperatively. Among the six patients, four were alive without tumors, one died due to lung metastasis and rupture of the tumor, and the children with a positive PRM died due to multiple retroperitoneal and pelvic recurrences without regular postoperative radiotherapy.

In group II, three patients experienced recurrence, and one patient died because of DDS-induced renal failure without recurrence. Of the three patients with recurrence, all underwent bilateral NSS, and the side of tumor recurrence in two patients was consistent with the side where the tumor was growing into the collecting system before surgery. Three patients with recurrence underwent surgical resection after tumor recurrence and additional chemotherapy was administered postoperatively. All the three patients were alive without tumors.

**Fig. 2:**
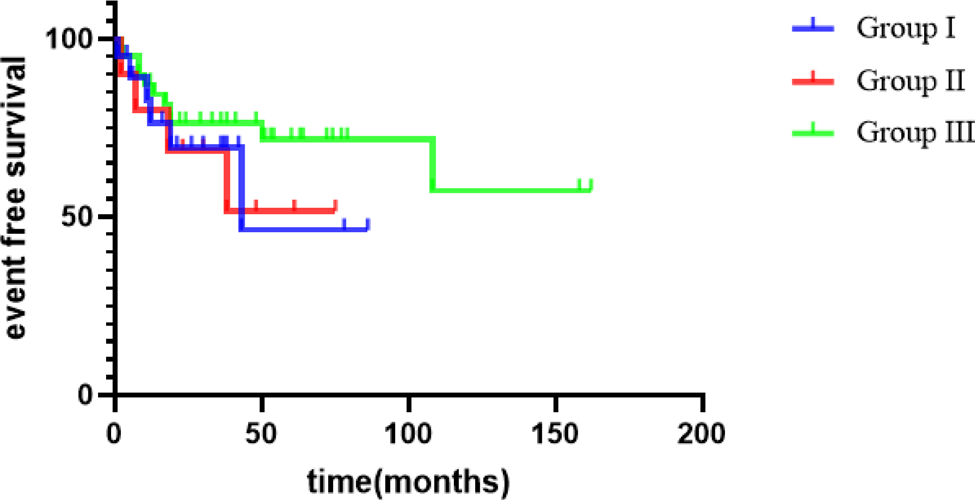
EFS survival curves of children with different BWT groups

**Fig. 3:**
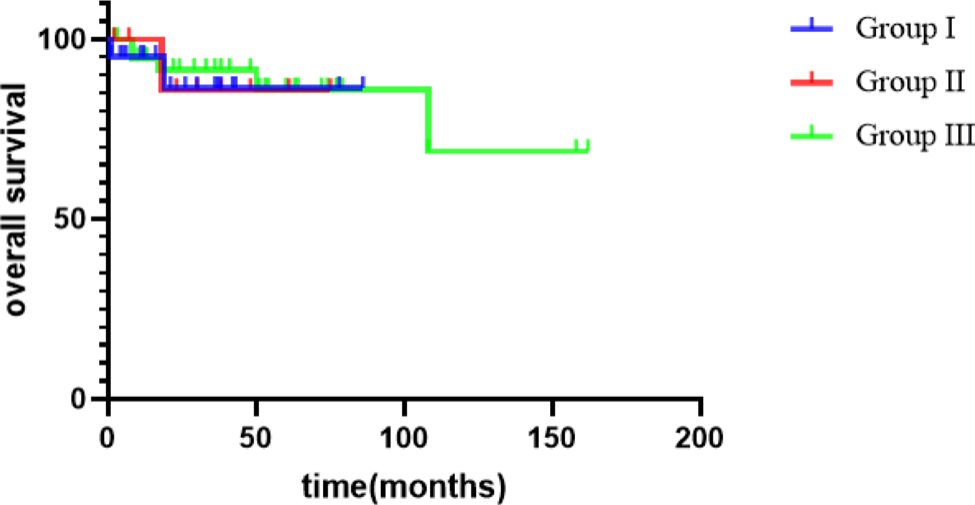
OS survival curve of children in different BWT groups

There was no significant difference in the 4-year EFS among groups I, II, and III (46.3%, 51.4%, and 76.2% respectively, *P* = 0.427), as shown in Fig. 2. The difference of 4-year EFS among group I plus II vs. group III was not statistically significant. Furthermore, there was no significant difference in the 4-year OS among groups I, II, and III (86.36%, 85.71%, and 91.40%, respectively, *P* = 0.902), as shown in Fig. 3.

## Discussion

In BWT when the tumor infiltrates or grows into the collecting system (botryoid growth pattern, BGP), total nephrectomy on one side and partial nephrectomy on the other increases the likelihood of long-term renal failure. The literature on NSS in children with BWT involving the collection system is mostly comprised of case reports. In the past 14 years, 70 patients with BWT were admitted to our center, including 10 patients with 13 kidney tumors (9.3%, 13/140) growing into the collecting system and 20 patients with 25 kidney tumors (17.9%, 25/140) infiltrating the collecting system. When the tumor involves the collecting system, the use of NSS is extremely challenging because of the need to protect the renal parenchyma and avoid intraoperative tumor rupture. Therefore, this study summarizes the clinical characteristics, treatments, and prognoses in these children.

BGP is a descriptive term used for WT with a lobular grape-like appearance growing in a polypoid manner into the renal pelvis and found in gross and histologic examinations in 10% of patients with WT [1]. In earlier publications, botryoid growth was referred to as a “rupture into the collecting system” [4, 5]. This description is misleading because BGP does not usually infiltrate the renal pelvic wall. The male-to-female ratio was 5:3 for BGP WT [6]. It has been reported that the common clinical manifestations of BGP WT are abdominal mass and hematuria [2]. In our study, in groups I and II, the main clinical manifestations were abdominal masses, accounting for 60% (18/30), followed by hematuria, accounting for 20%. The incidence of hematuria was higher in children with tumors involving the collecting system than in those without tumors involving the collecting system (20% vs. 7.5%, respectively), the difference was not significant, however, in clinical practice, if a child has hematuria, we should pay attention to the possibility of tumor involving the collection system.

CT scan can help to determine the relationship between tumor and collecting system. Because the BGP easily extends to the proximal ureter, hydronephrosis often appears on imaging. CT examination showed that the BGP mass was in the renal sinus area (imaging findings showed mass occupation in the renal pelvis and calyces) with an equal or slightly low-density shadow and that the renal parenchyma could be thinned due to pressure. If the densities are similar, the boundaries cannot be distinguished [7]. Tumors infiltrating the collecting system, renal pelvis, and calyces generally show deformation after compression on imaging, or an intrapelvic mass if the tumor invades the renal pelvis [5]. However, it seems that CT alone is not enough to identify collecting system involvement because only 13 children were identified by CT in our study. So relying on intraoperative findings remain the only diagnostic approach to determine whether a tumor has infiltrated the collection system.

The main reasons for performing a biopsy for preoperative chemotherapy in unilateral WT are to avoid misdiagnosis and confirm the presence of anaplasia. The pathological examination of groups I and II in our study showed no anaplasia. Our study findings were consistent with the Japan Wilms Tumor Study Group (JWiTS) series, which a pathological review revealed no cases of anaplastic BWT [8]. Therefore, our experience suggests that a routine preoperative biopsy is not needed before chemotherapy for BWT. In a prospective clinical trial, AREN0534, conducted by the COG, bilateral NSS was evaluated after 6 weeks of preoperative vincristine + actinomycin D + doxorubicin chemotherapy. If the tumor was reduced but bilateral NSS could not be performed, chemotherapy was continued for 12 weeks. If the tumor was stable or progressive, the chemotherapy regimen was adjusted to 12 weeks after bilateral biopsy before surgery [9]. The 4-year EFS and OS rates of BWT in that study were 82.1% and 94.9%, respectively, which were significantly higher than those reported by NWTS-5 (56.0% and 80.8%) [9]. In our study, in group I, 23 sides (57.5%) achieved PR, and in group II, 5 sides (27.8%) achieved PR. However, after preoperative chemotherapy, tumors in 13 kidneys grew into the collecting system and all patients had SD. This may be due to the inability of chemotherapy drugs to reach the renal pelvis, making the mass in the renal pelvis insensitive to chemotherapy drugs. However, it is worth noting that preoperative chemotherapy with the VA regimen was used in the early stage of this study, and doxorubicin can be further used to improve the effect of chemotherapy in the future.

The surgical approach to BWT may be complicated because tumor lesions need to be completely removed, sparing as much renal parenchyma as possible. Surgical management of BGP in BWT is challenging because BGP complicates NSS, especially the removal of the entire tumor and grape-like mass in the renal pelvis without rupture. In Li Z study, they analyzed the clinical and preoperative imaging data of BWT patients and identified three independent predictors for the feasibility of NSS, including tumor size, relationship with the collecting system, and residual renal parenchyma proportion [10]. Our study suggests that the boundary between the tumor and normal renal parenchyma should be accurately identified during NSS. Surgeons can predict the resection margin preoperatively with imaging preoperatively and reduce damage to important blood vessels and collection systems without affecting the tumor resection margin [11]. The renal pelvis and calyx should be carefully opened to excise the tumor. In our study, 11 patients (84.6%) underwent NSS, in which the entire tumor and grape-like mass in the pelvis were removed without rupture. Similarly, when a tumor infiltrates the collecting system, it is important to precisely identify the edge of the tumor. Part of the renal pelvic tissue can be excised to avoid positive tumor margins, and the collecting system can be carefully reconstructed. When a tumor infiltrates a large area of the renal pelvis and cannot be completely removed, it is necessary to administer stage III chemoradiotherapy after surgery to reduce tumor recurrence. In our study, 88% of the 25 kidneys with tumors involving the collecting system had NSS, and only one child was SRM-positive; NSS was performed when the tumor infiltrated the pelvis. The patient underwent radiotherapy and chemotherapy after surgery, and no recurrence was observed. Davidoff et al. reported no significant difference in the risk of local recurrence between children with positive and negative margins [12]. However, it also increases the risk of long-term postoperative hypertension [12]. In our study, one patient with a positive PRM did not receive regular radiotherapy, had a recurrence, and died. Complete tumor resection is feasible using NSS, as demonstrated by the fact that only one patient had margins infiltrated by the tumor. However, NSS is important for ensuring negative surgical margins.

BGP itself did not change the stage or prognosis of patients with WT. Vujanic et al. showed that BGP in the collecting system should not be a cause for elevating the tumor stage. Even if the tumor extends into the ureter and reaches the bladder without infiltrating the bladder wall, stage I should be considered if complete resection is performed. By contrast, wall infiltration should be regarded as stage II [1]. Shamberger et al. reported that the 10-year OS of patients with BWT treated with NWTS-4 was 82.1% [13]. In the JWiTS, the median follow-up time for 28 children with BWT was 8 years, the 5-year EFS was 85.5%, and the 5-year OS was 92.6% [8]. Sudour et al. reviewed the clinical data of 49 children with BWT treated according to the SIOP 93 guidelines in France, 67% of the kidneys underwent NSS, and the 5-year EFS and 5-year OS rates were 85.5% and 92.6%, respectively [14]. In our series, there was a significant difference in the prognosis of the 70 children with different tumor stages (92.3% vs. 64% vs. 50% vs. 40%); the 4-year EFS was 67.9%, and the 4-year OS was 89.3%. The OS rate was consistent with the literature reports, and the EFS was consistent with most of the literature reports but lower than that of AREN0534, which was thought to be related to the fact that preoperative chemotherapy mainly used vincristine and actinomycin and mostly did not add doxorubicin. There was no significant difference in the EFS among groups I, II, and III at 4 years (46.3% vs. 51.4% vs. 76.2%). There was no significant difference in the OS among groups I, II, and III at 4 years (86.36% vs. 85.71% vs. 91.40%, respectively). So a higher rate of events is present in BWT with collecting system involvement, but survival is good.

Important limitations of our study are its retrospective design and limited sample size. This was not a randomized controlled trial. Second, an insufficient description of the proportion of residual parenchyma is a limitation of this study. Third, we did not routinely use magnetic resonance imaging in our pediatric surgery center which in particular is internationally recommended. Moreover, the follow-up period in this study was short, and follow-up should be lengthened to monitor for tumor recurrence.

## Conclusions

Our study suggests that to preserve the renal parenchyma, NSS is feasible for children with BWT involving the collecting system. However, it should be noted that the intraoperative resection margin of the tumor was negative, and the tumor was completely removed. There was no significant difference in postoperative long-term OS between BWT involving the collecting system and BWT not involving the collecting system. It is the tumor stage itself, not the BGP, that influences prognosis.

## Data Availability

The datasets generated and/or analysed during the current study are available from Hong Cheng Song repository on reasonable request.

## Abbreviations

WT: Wilms’ tumors
BWT: Bilateral Wilms’ tumors
NSS: Nephron-sparing surgery
CT: Computed tomography
BGP: Botryoid growth pattern
CR: Complete response
PR: Partial response
PD: Progressive disease
SD: Stable disease
SIOP: International Society of Pediatric Oncology
EFS: Event-free survival
OS: Overall survival
SRM: Surgical resection margin
PRM: Pathological resection margin
NWTS/COG: National Wilms Tumor Study/Children’s Oncology Group
DDS: Denys–Drash syndrome
JWiTS: Japan Wilms Tumor Study Group
